# *clifford ^B.4.1^* , an allele of *CG1603* , causes tissue overgrowth in the *Drosophila melanogaster* eye

**DOI:** 10.17912/micropub.biology.000936

**Published:** 2023-08-22

**Authors:** Reagan R Nowaskie, Ashley Kitch, Abby Adams, Abinaya Anandaraj, Ethan Apawan, Liliana Bañuelos, Cassandra J Betz, Julia M Bogunia, Nicholas Buechlein, Morgan R Burns, Hayley A Collier, Zach Collins, Kynzie Combs, Vana D Dakarian, Abigail Daniel, Conrad M De Jesus III, John D Erickson, Bianca Estrada, Kevin Estrada, Sydney Fields, Maya Gabriel, Rosario M Garcia, Sylvia Gitamo, Emma Granath, Sabrina N Hardin, Emily Hattling, Alexandra VL Henriquez, Destiny Hernandez, Luke Johnson, Annie H Kim, Lillian K Kolley, Katelynn M Larue, Erin Lockwood, Nelia Longoria, Cassandra Lopez, Rosario C Lopez-Roca Fernandez, Sofia Lozano, Carissa Manthie, Trinity May, Zorah Mehrzad, Itzel Mendoza, Somya Mohan, Claylan Mounthachak, Merveille Muyizere, Margaret R Myers, Jayce Newton, Amarachi Nwawueze, Ariana J Paredes, Marissa N Pezdek, Hoang Phat Nguyen, Nadia Pobuda, Sahar Sadat, Johnathon J Sailor, David Santiago, Madison Sbarbaro, David E Schultz III, Anahita N Senobari, Emma M Shouse, Sarah M Snarski, Estefanie Solano, Naomi Solis Campos, Elnora Stewart, Jessica Szczepaniak, Michael Tejeda, Dominic F Teoli, Michael Tran, Nishita Trivedi, Laurita Uribe Aristizabal, Bryan Z Vargas, Kenneth W Walker III, Joseph Wasiqi, Joyi Wong, Adira Zachrel, Hemin P Shah, Elizabeth Small, Charlie T Watts, Paula Croonquist, Olivier Devergne, Amy K Jones, Elizabeth E Taylor, Jacob D Kagey, Julie A Merkle

**Affiliations:** 1 University of Evansville, Evansville, Indiana, United States; 2 Northern Illinois University, DeKalb, Illinois, United States; 3 Anoka-Ramsey Community College, Coon Rapids, Minnesota, United States; 4 The University of Texas at San Antonio, San Antonio, Texas, United States; 5 University of Detroit Mercy, Detroit, Michigan, United States

## Abstract

Mutant *B.4.1* , generated via EMS mutagenesis in *Drosophila* *melanogaster* , was studied by undergraduate students participating in the Fly-CURE. After inducing genetically mosaic tissue in the adult eye, *B.4.1* mutant tissue displays a robust increase in cell division and a rough appearance. Complementation mapping and sequence analysis identified a nonsense mutation in the gene *CG1603* , which we named *clifford* ( *cliff* ) due to observed increases in red-pigmented mutant tissue compared to controls. *cliff* encodes a zinc finger-containing protein implicated in transcriptional control. RNAi knockdown of *cliff* similarly results in rough eyes, confirming a role for Cliff in eye development.

**Figure 1. Characterization of the cliff B.4.1 mutation by phenotypic analysis, complementation mapping, and genetic sequencing:**
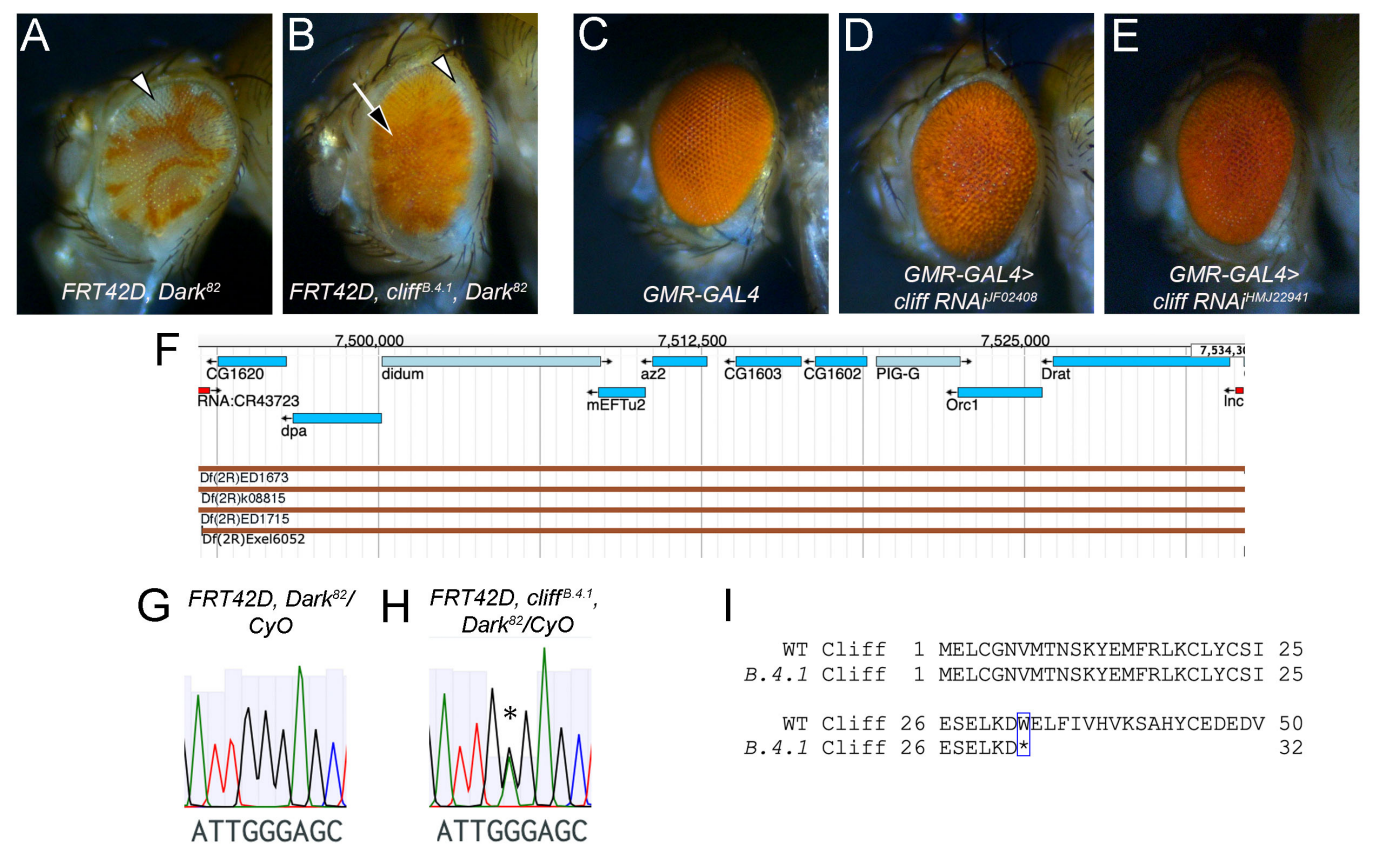
**(A-B)** The FLP/FRT genetic system was used to induce mitotic recombination in the adult *Drosophila* eye to examine the *B.4.1* mutant phenotype compared to controls. After mitotic recombination, control eyes ( **A** , genotype *ey>Flp* , *w ^–^ ; FRT42D, Dark ^82^ /FRT42D* ) exhibited a higher percentage of wildtype non-pigmented tissue (arrowhead) compared to eyes of *B.4.1* mutant flies ( **B** , arrowhead; genotype *ey>Flp* , *w ^–^ ; FRT42D* , *cliff ^B.4.1^* , *Dark ^82^* / *FRT42D* ), which displayed nearly entirely pigmented mutant tissue ( **B,** arrow). **(C-E)** RNAi knockdown of *CG1603* with *GMR-GAL4* results in rough eyes, phenocopying *cliff ^B.4.1^* mutant clones in the eye. **(C)** *GMR-GAL4* control eye. **(D-E)** Two independent RNAi lines targeting *CG1603* ( *cliff* ) result in rough eyes under the control of *GMR-GAL4* . **(F)** Map of chromosome 2R showing the deficiency lines (red bars) that failed to complement the *B.4.1* mutation and yielded a region of overlap at chromosomal location 2R:7493197..7533553. Image adapted from JBrowse on FlyBase, release FB2023_03 (Gramates et al. 2022). **(G-H)** The nucleotide locus of the *B.4.1* mutation was identified by Sanger sequence analysis. **(G)** The control sequence (genotype *w ^–^ ; FRT42D, Dark ^82^ /CyO* ) contained a single G peak at position 2R:7,516,167, which is within the coding region of *CG1603* . **(H)** The mutant sequence (genotype *w ^–^ ; FRT42D* , *cliff ^B.4.1^* , *Dark ^82^ /CyO* ) contained a heterozygous peak revealing a nucleotide change from G to A at this position (*). **(I)** Alignment of wildtype (WT) and *B.4.1* mutant Clifford (Cliff) protein. The nonsense mutation (*) identified in the *B.4.1* mutant line at amino acid 32 (W, tryptophan in wildtype) is indicated (blue box).

## Description

The Fly-CURE project uses *Drosophila melanogaster* and genetic techniques to uncover genes involved in cell growth control that could aid in the understanding of cancer development and progression (Merkle et al. 2023) . The *B.4.1* mutation was generated by an EMS mutagenesis screen in *Drosophila* . The FLP-FRT system was used to induce mitotic recombination on the right arm of chromosome 2 (2R), thereby producing *B.4.1* homozygous mutant cells in the adult *Drosophila* eye (Kagey et al. 2012) . To allow for the characterization of genes involved in cell growth whose function could be masked by apoptosis, this process was prevented using a mutant allele of the gene *Death-associated APAF-related killer* ( *Dark* ; mutant allele, *Dark ^82^* ), which is required for apoptosis, evolutionarily conserved, and homozygous lethal when mutant (Rodriguez et al. 1999) . The *Dark ^82^* mutation is due to a transposon insertion carrying a *mini* - *white* cassette that produces red pigment in the *Drosophila* eye. Mitotic recombination results in homozygous mutant patches of cells only within the eye. *B.4.1* and *Dark ^82^* are both on chromosome 2R and therefore inherited together after mitotic recombination, distinguishing mutant cells (pigmented, red cells) from wild-type cells (non-pigmented, white cells).

Phenotypic analysis of the *B.4.1* mutation was carried out by crossing flies that carry FLP recombinase controlled by the *eyeless* ( *ey* ) promoter ( *ey>FLP* ) and an FRT site on chromosome arm 2R ( *FRT42D* ) with flies that carry the *B.4.1* mutation (𝑤 ^−^ ; *FRT42D, Dark ^82^ , B.4.1/CyO* ) as the experimental group, or flies lacking the *B.4.1* mutation (𝑤 ^−^ ; *FRT42D, Dark ^82^ /CyO* ) as a control group. The resulting progeny of the crosses were examined for phenotypically mosaic eyes. The *B.4.1* mutation resulted in mosaic eyes with significantly more mutant tissue, with an average of 91% red to 9% white tissue (n=80) ( **Figure 1B** , arrowhead denotes non-pigmented wildtype tissue and arrow indicates pigmented mutant tissue). In comparison, the control group displayed an average of 54% red to 46% white tissue (n=140) ( **Figure 1A** , arrowhead denotes non-pigmented wildtype tissue). Interestingly, it appeared that heterozygous and homozygous mutant tissue could be observed in the *B.4.1* mutant eyes, displaying 35% orange heterozygous tissue and 65% red homozygous mutant tissue (n=80). Furthermore, *B.4.1* mutant eyes appeared rough with misaligned ommatidia compared to control eyes. These data suggest that the gene affected by the *B.4.1* mutation is involved in proper eye development and organization. Tissue overgrowth was confined to the eye and not seen in the extraocular region, suggesting the effects of the *B.4.1* mutation are cell autonomous. Since the observed overgrowth of red-pigmented mutant tissue was reminiscent of “Clifford the Big Red Dog”, the gene affected by the *B.4.1* mutation was named *clifford* ( *cliff* ).

Genetic complementation mapping was performed by crossing *cliff ^B.4.1^* mutant females with males from individual lines in the Bloomington *Drosophila* Stock Center 2R Deficiency Kit (Cook et al. 2012) . At least fifty progeny from each cross were counted and evaluated for complementation status based on survival or lethality of transheterozygous progeny. If the progeny had curly and straight wings, the deficiency and mutant chromosomes were determined to complement each other. If the progeny had only curly wings, the deficiency and mutant chromosomes were determined to fail to complement each other. Deficiency lines *Df(2R)ED1715/SM6a* and *Df(2R)ED1673/SM6a* failed to complement the *cliff ^B.4.1^* mutant chromosome ( **Table 1** and **Figure 1F** ), narrowing the location of the *cliff ^B.4.1^* mutation to 2R:7,326,951..7,533,553. Another region that failed to complement the mutant chromosome was eliminated as the *cliff ^B.4.1^* locus because it contains the *Dark* gene and served as positive controls for complementation mapping. Subsequently, smaller deficiencies in the region of overlap were tested. Two additional deficiency lines, *Df(2R)Exel6053/CyO* and *Df(2R)k08815/CyO* , failed to complement the *cliff ^B.4.1^* mutation, indicating that the *cliff ^B.4.1^* mutation is within the region 2R:7,493,197..7,533,553 ( **Table 1** ). Mutant alleles for individual candidate genes ( *CG1603* , *dilute class unconventional myosin* ( *didum* ), *disc proliferation abnormal* ( *dpa* ), and *Origin recognition complex subunit 1* ( *Orc1* )) were then tested by complementation. A mutant allele of *CG1603* ( *CG1603 ^f04743^* ) failed to complement the *cliff ^B.4.1^* mutant chromosome, suggesting that *cliff ^B.4.1^* is an allele of *CG1603* , which encodes a predicted zinc finger-containing protein that interacts with RNA polymerase II during transcriptional control (Ashton-Beaucage et al. 2014) . Since the protein-coding gene *CG1603* is not yet named in *Drosophila* , the name *clifford* ( *cliff* ) is proposed to reflect the recessive phenotype observed in mutant eye clones.

**Table 1. Complementation results using the mutant *cliff ^B.4.1^* and deficiency lines or single gene alleles on chromosome 2R.** Complementation results with chromosome 2R deficiency lines that failed to complement the *cliff ^B.4.1^* mutant chromosome, narrowing the location of the *cliff ^B.4.1^* mutation to 2R:7,326,951..7,533,553. *Dark* is located at 2R:17,020,027..17,026,703 and the *Dark ^82^* mutation is inherited with the *cliff ^B.4.1^* mutation, thereby eliminating this locus as the *cliff ^B.4.1^* region. Additional deficiency lines narrowed the *cliff ^B.4.1^* locus to 2R:7,493,197..7,533,553. Individual alleles of candidate genes in this region were then tested by complementation analysis with *cliff ^B.4.1^ ;* an allele of *CG1603* failed to complement the *cliff ^B.4.1^* mutant line.

**Table:** 

** Bloomington *Drosophila* Stock Center (BDSC) 2R Deficiency Kit **			
**Deficiency**	**BDSC Stock #**	**Region**	**Complementation Result**
*Df(2R)ED1673*	9062	6,985,802..7,533,553	Fail to complement
*Df(2R)ED1715*	8931	7,326,951..7,916,923	Fail to complement
*Df(2R)ED2747*	9278	16,829,073..17,097,303	Fail to complement
*Df(2R)BSC331*	24356	16,869,330..17,139,923	Fail to complement
**Additional Deficiency Lines**			
*Df(2R)BSC263*	23162	7,146,864..7,447,410	Complement
*Df(2R)BSC264*	23163	7,395,885..7,489,834	Complement
*Df(2R)k08815*	10818	7,489,834..7,665,893	Fail to complement
*Df(2R)Exel6052*	7534	7,493,197..7,623,083	Fail to complement
*Df(2R)Exel6053*	7535	7,533,553..7,665,795	Complement
**Individual Gene Alleles**			
**Genotype**	**BDSC Stock #**	**Gene of interest**	**Complementation Result**
* CG1603 ^f04743^ /CyO *	18801	*CG1603*	Fail to complement
* didum ^KG04384^ /CyO *	14094	*dilute class unconventional myosin (didum)*	Complement
* dpa ^1^ bw ^D^ /CyO *	4126	*disc proliferation abnormal (dpa)*	Complement
* Orc1 ^KO^ /CyO *	77867	*Origin recognition complex subunit 1 (Orc1)*	Complement

PCR and Sanger sequence analysis revealed a nucleotide change at 2R:7,516,170 in the *CG1603* gene in *cliff ^B.4.1^* heterozygous flies. The *Dark ^82^* control sequence showed a single G peak at this position **( Figure 1G )** , while the *cliff ^B.4.1^* mutant sequence showed two peaks (G and A) at this locus **( Figure 1H** , asterisk **)** and was confirmed by two independent PCR primer pairs and resulting sequencing reads. This G to A change in the *cliff ^B.4.1^* mutant sequence results in a nonsense mutation at amino acid 32 of the polypeptide produced by *CG1603* , conferring a premature stop codon instead of tryptophan in the wild-type protein **( Figure 1I )** .

To confirm the role of *cliff* in eye development, RNAi knockdown was performed using the eye-specific GAL4 driver *GMR-GAL4* . Two independent RNAi lines for *cliff* generated by the Transgenic RNAi Project (TRiP) ( **Figure 1D** , JF02408; **Figure 1E** , HMJ22941) were used and compared to GAL4-only control flies. Adult eyes expressing *cliff* RNAi under the control of *GMR-GAL4* displayed rough eyes with misaligned ommatidia compared to control ommatidia displaying an expected highly organized pattern ( **Figure 1C** ). These data validate the phenotype observed in *cliff ^B.4.1^* mutant clones, supporting the role of *cliff* in *Drosophila* eye development and organization.

CG1603, newly named Clifford (Cliff), contains zinc finger domains and is predicted to function in DNA binding and transcription (Ashton-Beaucage et al. 2014) . Based on the observed phenotype and identified nonsense mutation, the *cliff ^B.4.1^* mutation likely results in Cliff loss-of-function, which may normally function to regulate the binding of machinery necessary for proper transcription. Without Cliff function, an overgrowth of mutant tissue results, yielding the observed mutant phenotype. A previous study showed that RNAi knockdown of *cliff* results in reduced *mapk* transcription and suppression of the *sev-Ras ^V12^ /+* rough eye phenotype in *Drosophila* (Ashton-Beaucage et al. 2014) . Cliff has also been shown to interact with Apoptosis inducing factor (AIF) (Guruharsha et al. 2011) , which is involved in the development of the eye imaginal disc by regulating apoptosis in *Drosophila* (Joza et al. 2008) . Future studies are required to uncover the precise role(s) of Cliff and the molecular pathway(s) that it is involved in during eye development.

The predicted human orthologs of *cliff* also encode zinc finger proteins, which generally are responsible for a wide array of molecular functions by interacting with RNA, DNA, and/or other proteins (Cassandri et al. 2017) . If a zinc finger-containing protein functions to suppress the transcription of genes involved in promoting cell growth or division by acting as a putative tumor suppressor, or if it functions to promote the transcription of genes involved in preventing cell growth or division by acting as a putative proto-oncogene, a loss-of-function mutation could lead to uncontrolled cell growth, potentially leading to the development of cancer. Further investigation of Cliff could lead to a better understanding of the role(s) of its human orthologs in cancer development and progression.

## Reagents

*w ^–^ ; FRT42D, Dark ^82^ /CyO* (Akdemir et al. 2006)

*w ^–^ ; FRT42D, Dark ^82^ , B.4.1/CyO* (this study)

*w ^–^ , ey>FLP; FRT42D* (BDSC 5616)

Bloomington Drosophila Stock Center 2R Deficiency Kit (Cook et al. 2012)

*w ^1118^ ; Df(2R)BSC263/CyO* (BDSC 23162)

*w ^1118^ ; Df(2R)BSC264/CyO* (BDSC 23163)

*w ^1118^ ; Df(2R)Exel6052, P{w ^+mC^ =XP-U}Exel6052/CyO* (BDSC 7534)

*w ^1118^ ; Df(2R)Exel6053, P{w ^+mC^ =XP-U}Exel6053/CyO* (BDSC 7535)

*y ^1^ w ^67c23^ ; Df(2R)k08815, P{w ^+mC^ =lacW}wech ^k08815^ /CyO* (BDSC 10818)

*Dp(?;2)bw ^D^ , dpa ^1^ bw ^D^ /CyO* (BDSC 4126)

*y ^1^ ; P{y ^+mDint2^ w ^BR.E.BR^ =SUPor-P}didum ^KG04384^ /CyO* (BDSC 14094)

w*; TI{w ^+mC^ =TI}Orc1 ^KO^ /CyO, P{w ^+mC^ GAL4-Kr.C}DC3, P{w ^+mC^ =UASGFP.S65T}DC7 (BDSC 77867)

*w ^1118^ ; PBac{w ^+mC^ =WH}CG1603 ^f04743^ /CyO* (BDSC 18801)

*y ^1^ v ^1^ ; P{y ^+t7.7^ v ^+t1.8^ =TRiP.JF02408}attP2* (BDSC 27063)

*y ^1^ v ^1^ ; P{y ^+t7.7^ v ^+t1.8^ =TRiP.HMJ22941}attP40* (BDSC 61220)

w*; P{w ^+mC^ = GAL4-ninaE.GMR}12 (BDSC 1104)

*CG1603* forward primer 1: 5’ CAA GCT GAA GTC AGA TCA GAG C 3’

*CG1603* reverse primer 1: 5’ CTG AAT GAC GCC GTT AAG TGG 3’

*CG1603* forward primer 2: 5’ ATG GAG CTG TGC GGC AAT GTG AT 3’

*CG1603* reverse primer 2: 5’ CCA TCC ATT GAT CGA CCC CAT TGC 3’
